# Fractal Analysis of Jaw Bone Structure in Individuals With Dental Fluorosis: A Retrospective Cross‐Sectional Study

**DOI:** 10.1002/cre2.70480

**Published:** 2026-10-12

**Authors:** Ozlem Gormez, Hasan Huseyin Yilmaz, Abdulkadir Cakir

**Affiliations:** ^1^ Faculty of Dentistry, Department of Oral and Maxillofacial Radiology Süleyman Demirel University Isparta Turkey; ^2^ Independent dentist Isparta Turkey; ^3^ Faculty of Technology, Department of Electrical‐Electronics Engineering Isparta University of Applied Sciences Isparta Turkey

**Keywords:** fluorosis, fractal analysis, image processing, jaw

## Abstract

**Objectives:**

This retrospective cross‐sectional study aimed to evaluate differences in maxillary and mandibular trabecular bone structure in individuals with dental fluorosis using fractal dimension (FD) analysis.

**Materials and Methods:**

The study included 47 individuals: 24 with dental fluorosis and 23 controls. Fluorosis severity was assessed using the Thylstrup–Fejerskov Index and classified as moderate (*n* = 13) or severe (*n* = 11). FD analysis was performed on standardized regions of interest on digital panoramic and digitized analog periapical radiographs using ImageJ. Normality was assessed using the Shapiro–Wilk test. Region‐specific FD values were compared using independent‐samples *t*‐tests or Mann–Whitney *U* tests, as appropriate. Generalized estimating equation (GEE) analyses were used to account for within‐participant correlation among repeated regional measurements.

**Results:**

In region‐specific comparisons, FD was significantly lower in the fluorosis group than in controls in the 33–34 panoramic region (1.46 ± 0.04 vs. 1.48 ± 0.03; *p* = 0.048). FD was significantly higher in severe than moderate fluorosis in the 13–14 panoramic region (1.36 ± 0.05 vs. 1.28 ± 0.06; *p* = 0.004) and the 11–12 periapical region (1.63 ± 0.01 vs. 1.61 ± 0.02; *p* = 0.019). GEE analysis showed a significant average marginal fluorosis‐control difference for panoramic radiographs (mean difference = −0.0210, 95% CI: −0.0386 to −0.0034; *p* = 0.020), without a significant group‐by‐region interaction (*p* = 0.840). For fluorosis severity, the severity‐by‐region interaction was significant for panoramic radiographs (*p* = 0.004), while average marginal periapical FD was higher in the severe subgroup (*p* = 0.028).

**Conclusions:**

FD analysis identified differences in radiographic trabecular pattern complexity associated with dental fluorosis, although findings varied by anatomical region and imaging modality. Given the small sample, multiple regional comparisons, and moderate reproducibility of periapical measurements, these findings should be considered exploratory and require confirmation in larger studies using standardized imaging protocols.

## 1 Introduction

Fluoride is a natural element with systemic effects on the human body and is considered physiologically essential for normal growth and development, particularly for tooth and bone formation. At appropriate levels, its effectiveness in preventing dental caries has been proven, and it is widely used in dental health applications (Kaminsky et al. 1990; Dhar and Bhatnagar 2009; Chachra et al. 2010; Buyukkaplan and Guldag 2012; Hung et al. 2023; Veneri et al. 2023). However, since the safety margin of fluoride is relatively narrow, excessive exposure may lead to acute and chronic toxic effects (Kaminsky et al. 1990; Dhar and Bhatnagar 2009; Veneri et al. 2023; Tamer et al. 2007; Han et al. 2021; Choubisa 2022; Gezahegn and de Kiev 2023). Humans are exposed to fluoride through various sources such as drinking water, certain foods (particularly black tea, seafood, and some processed foods), oral care products, and inhaled air. Fluoride is mostly absorbed from the gastrointestinal tract; although a portion of it is excreted in the urine, a considerable amount is deposited in calcified tissues such as bones and teeth (Kaminsky et al. 1990; Dhar and Bhatnagar 2009; Chachra et al. 2010; Buyukkaplan and Guldag 2012; Veneri et al. 2023; Choubisa 2022; Kabir et al. 2019). Excess intake of fluoride beyond the recommended daily dose leads to chronic toxicity, resulting in a condition known as fluorosis. When affecting the teeth, the condition is referred to as dental fluorosis, and when seen in bones, it is defined as skeletal fluorosis; both are significant health problems that can cause permanent changes (Kaminsky et al. 1990; Hung et al. 2023; Veneri et al. 2023; Han et al. 2021; Choubisa 2022; Gezahegn and de Kiev 2023).

Fluorosis is endemic in several regions of the world where groundwater contains naturally high fluoride concentrations, and the province of Isparta in Turkey is among these areas (Kaminsky et al. 1990; Buyukkaplan and Guldag 2012; Veneri et al. 2023; Tamer et al. 2007; Kabir et al. 2019; Savas et al. 2001; Yildiz et al. 2003). While dental fluorosis is primarily an aesthetic concern, skeletal fluorosis may lead to alterations in bone metabolism and structure. Fluorosis‐related enamel defects initially appear as white or yellowish spots, which may gradually darken to brown discolorations over time. In advanced cases, marked pitting and brittleness of the teeth may be observed. While dental fluorosis occurs only during tooth development as a result of fluoride exposure, skeletal fluorosis can develop at any age because bone tissue undergoes continuous remodeling throughout life (Kaminsky et al. 1990; Dhar and Bhatnagar 2009; Hung et al. 2023; Veneri et al. 2023; Han et al. 2021; Gezahegn and de Kiev 2023). Early stages of skeletal fluorosis may not present with obvious clinical symptoms but can be detected radiographically through changes in bone density and architecture. As the condition progresses, it can lead to serious clinical outcomes that affect general health. Therefore, studies investigating skeletal fluorosis are of great importance for both early diagnosis and public health (Dhar and Bhatnagar 2009; Veneri et al. 2023; Choubisa 2022; Wang et al. 1994). Since craniofacial bones may be affected by systemic conditions, routine dental radiographs may provide valuable information about bone structure (Pachêco‐Pereira et al. 2019). Advances in digital imaging have enabled the application of image processing techniques that allow both qualitative and quantitative evaluation of trabecular bone patterns (Yaşar and Akgünlü 2005; Tosoni et al. 2006). One of these techniques, fractal analysis (FA), is a mathematically based texture evaluation method that quantitatively characterizes the structural complexity of irregular patterns. Within this analysis, the fractal dimension (FD), calculated through computer‐based algorithms, expresses the geometric complexity of a structure as a single numerical value (Pachêco‐Pereira et al. 2019; Tosoni et al. 2006; Geraets and van der Stelt 2000; Bollen et al. 2001; Binici and Özbay Çetiner 2024). FD analysis is a quantitative image‐processing technique that evaluates the complexity of trabecular bone architecture using routine dental radiographs without requiring additional radiation exposure. Therefore, it represents a suitable method for investigating subtle bone alterations that may not be detectable by conventional radiographic assessment alone. FA has been widely used in dentistry to assess trabecular bone structure under different systemic and local conditions, particularly osteoporosis (Geraets and van der Stelt 2000; Binici and Özbay Çetiner 2024; White and Rudolph 1999; Yaşar and Akgünlü 2006; Geraets et al. 2008).

More recently, FD analysis has been increasingly applied in various areas of dentistry, including systemic conditions affecting bone metabolism, orthodontic treatment, implant dentistry, craniofacial growth, and other oral and congenital disorders. Higher FD values generally reflect greater trabecular pattern complexity, whereas trabecular loss or structural disruption is associated with lower FD values (Pachêco‐Pereira et al. 2019; Yaşar and Akgünlü 2006; Kangvansurakit 2017; Çiçek et al. 2024; Özden and Cicek 2024; Cicek et al. 2025). Recent reviews indicate that FD can provide a quantitative measure of the complexity of trabecular patterns in dental images; however, substantial methodological heterogeneity remains regarding ROI selection, image processing, and FD calculation, and standardized diagnostic thresholds have not yet been established (Franciotti et al. 2021; da Silva et al. 2023; Colaco et al. 2025). Previous radiological studies have mainly focused on descriptive radiographic findings or bone mineral density measurements in skeletal fluorosis, whereas quantitative assessment of jawbone trabecular microarchitecture has remained unexplored (Buyukkaplan and Guldag 2012; Gezahegn and de Kiev 2023; Yildiz et al. 2003; Wang et al. 1994). Therefore, the present study was designed to address this methodological gap by quantitatively evaluating jawbone trabecular structure using FD analysis on panoramic and periapical radiographs. Both panoramic and periapical radiographs were included because they are routinely used in dental practice, readily available, cost‐effective, and involve relatively low radiation exposure. Although both provide two‐dimensional representations of trabecular bone and are subject to image distortion and superimposition, they remain suitable for quantitative image‐processing methods such as FD analysis. The selected anatomical regions predominantly contain trabecular bone and have been commonly used in previous FD studies, and allow standardized quantitative comparisons. The aim of this retrospective cross‐sectional study was to quantitatively compare FD values obtained from predefined maxillary and mandibular trabecular regions on panoramic and periapical radiographs between individuals with dental fluorosis and healthy controls, as well as between moderate and severe fluorosis subgroups. It was hypothesized that individuals with dental fluorosis would exhibit differences in FD values in selected jaw regions compared with individuals without dental fluorosis, and that FD values would differ according to fluorosis severity.

## 2 Materials and Methods

### 2.1 Study Design and Participants

This retrospective cross‐sectional study included individuals who presented to the Department of Oral and Maxillofacial Radiology, Faculty of Dentistry, Suleyman Demirel University, for various reasons. Participants were eligible for inclusion if they had complete clinical records and radiographic examinations of adequate quality for FD analysis. The fluorosis group consisted of 24 individuals clinically diagnosed with dental fluorosis, while the control group consisted of age‐ and sex‐matched individuals without clinical signs of dental fluorosis who had not been born or raised in the central region of Isparta. The sample size was determined based on the number of patients with dental fluorosis who met the inclusion criteria and presented to the clinic during the study period. Because dental fluorosis cases meeting the eligibility criteria were relatively limited in the study region, all eligible individuals presenting during the study period were included. The medical histories of all participants were reviewed to identify systemic diseases or treatments that could affect bone metabolism. Participants with systemic conditions affecting bone metabolism, a history of bone malignancy, or receiving medications known to adversely affect bone metabolism were excluded. Participants with inadequate radiographic image quality that precluded reliable ROI selection and FD analysis were also excluded. Ethical approval was obtained from the Ethics Committee of Suleyman Demirel University, Faculty of Medicine (Date/Decision no: 2011/03‐11), and written informed consent was obtained from all participants before inclusion in the study. The study consisted of 24 individuals (12 females and 12 males) with dental fluorosis who were born and raised in Isparta, and 23 individuals (14 females and 9 males) without dental fluorosis who were not born or raised in Isparta. The mean age of the study group was 35.04 ± 11.54 years, and that of the control group was 37.91 ± 11.35 years. Dental fluorosis was classified according to the Thylstrup‐Fejerskov Index (TFI) (Fejerskov et al. 1996). Before TFI assessment, the examiner was calibrated using a reference image illustrating the different TFI scores. To standardize the assessment conditions, intraoral photographs were obtained under controlled lighting conditions in a darkened room using daylight illumination. Frontal views and right and left lateral views were obtained using an intraoral mirror. TFI scoring was subsequently performed using these standardized photographic records. The highest TFI score observed for each participant was used for classification of fluorosis severity. The fluorosis group was divided into two subgroups: moderate fluorosis (TFI 5–6, *n* = 13) (Figure 1), and severe fluorosis (TFI 7–8, *n* = 11) (Figure 2).

**Figure 1:**
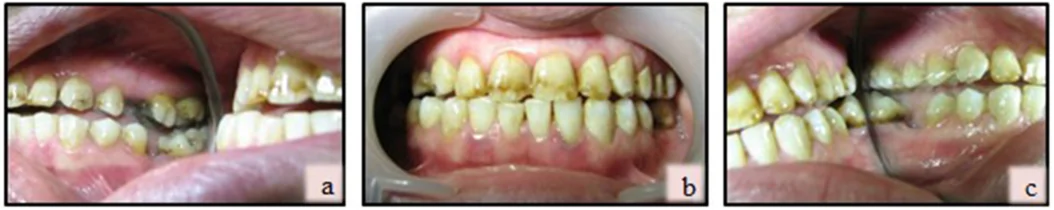
Intraoral photographs of a patient with moderate dental fluorosis. (a) Mirror image of the buccal surfaces of the right maxillary and mandibular posterior teeth, (b) Front view of the maxillary and mandibular anterior teeth, (c) Mirror image of the buccal surfaces of the left maxillary and mandibular posterior teeth.

**Figure 2:**
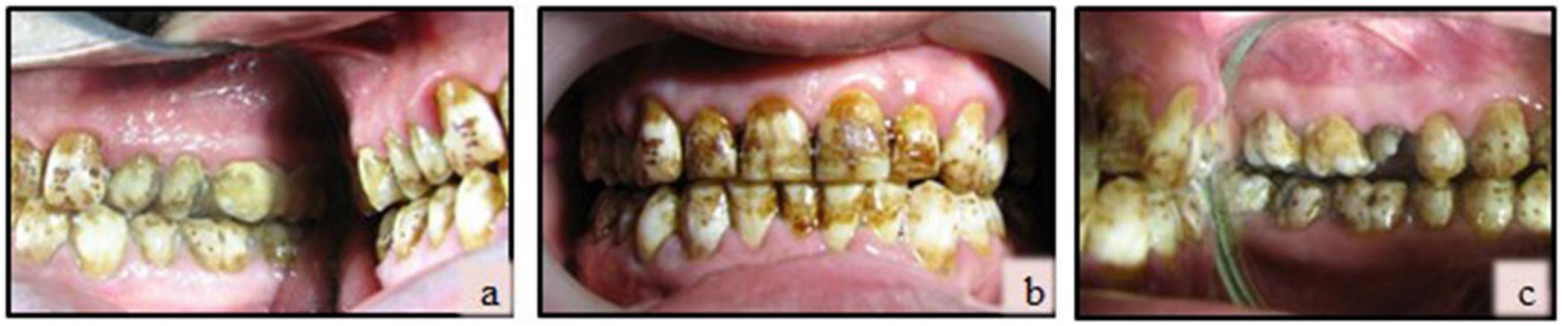
Intraoral photographs of a patient with severe dental fluorosis. (a) Mirror image of the buccal surfaces of the right maxillary and mandibular posterior teeth, (b) Front view of the maxillary and mandibular anterior teeth, (c) Mirror image of the buccal surfaces of the left maxillary and mandibular posterior teeth.

### 2.2 Image Acquisition

Panoramic radiographs provide a comprehensive evaluation of both jaws, whereas periapical radiographs provide higher spatial resolution for localized trabecular bone assessment. Panoramic and periapical radiographs were evaluated in this study because both are routinely used in dental practice and provide complementary information regarding trabecular bone structure. Digital panoramic radiographs were obtained using a panoramic imaging unit (Planmeca Proline PM2002CC, Helsinki, Finland, 66kVp and 8 mA). Additionally, analog periapical radiographs were obtained from the maxillary and mandibular central incisor regions (Gendex Oralix A. C. Densomat, Milan, Italy, Kodak E speed 2 film, 65 kVp and 7.5 mA, 0.26 s). All radiographs had been acquired according to the manufacturer's standard positioning protocol as part of routine clinical practice. All periapical films were processed using an automatic processor (Dürr XR 24, Germany) and subsequently digitized with a scanner equipped with a transparency adapter (Epson Perfection V700). Scanning features were set to be standard for all images, with 8‐bit gray value and 600dpi resolution (32 × 42 mm/755 × 992pixels), thereby minimizing variability attributable to digitization. This resolution has been widely used in previous FD studies. All images were saved in TIFF format with automatic corrections disabled. These settings were selected to preserve image detail while maintaining consistency across all digitized images.

### 2.3 ROI Selection and FD Analysis

Although panoramic radiographs were acquired digitally and periapical radiographs were digitized from conventional films, all images were converted to standardized 8‐bit grayscale images before FD analysis. FD analysis was performed on the same computer (Asus K56CM) using ImageJ 1.46r software (National Institutes of Health, USA; http://rsbweb.nih.gov/ij/, ImageJ 1.46r, NIH Image, Retrieved June 11, 2013). Regions of interest (ROIs) were selected from trabecular bone areas while avoiding tooth roots and anatomical structures and were positioned as apically as possible relative to the alveolar crest. On panoramic radiographs; 25 × 35 pixels ROI was placed in the trabecular bone between the maxillary right canine‐premolar (13–14) region, 25 × 35 pixels ROI was placed in the trabecular bone between the maxillary left canine‐premolar (23–24) region, 50 × 70 pixels ROI was placed in the trabecular bone between mandibular right canine‐premolar (43–44) region, 50 × 70 pixels ROI was placed in the trabecular bone between mandibular left canine‐premolar (33–34) region (Figure 3). On periapical radiographs; 50 × 50 pixels ROI was placed in the trabecular bone between the maxillary right central‐lateral incisor (11–12) region, 50 × 50 pixels ROI was placed in the trabecular bone between the maxillary left central‐lateral incisor (21–22) region, 50 × 50 pixels ROI was placed in the trabecular bone between the mandibular right central‐lateral incisor (41–42) region, 50 × 50 pixels ROI was placed in the trabecular bone between the mandibular left central‐lateral incisor (31–32) region (Figure 3). ROI placement was standardized using adjacent anatomical landmarks, particularly the apical region and the area away from tooth roots, to ensure reproducibility. Although ROI dimensions differed between panoramic and periapical radiographs because of differences in image resolution and anatomical coverage between the two imaging modalities, the same ROI dimensions and anatomical criteria were consistently applied to corresponding regions within each modality and across all participants. Different ROI sizes were selected to accommodate anatomical differences and image resolution between panoramic and periapical radiographs while ensuring that each ROI contained predominantly trabecular bone. Although ROI dimensions differed between imaging modalities, the dimensions were standardized within each modality and consistently applied to corresponding anatomical regions across participants. The ROIs were processed with standard image processing steps according to the method described by White and Rudolph (White and Rudolph 1999). Briefly, each ROI was duplicated and processed using Gaussian blur (sigma = 35), subtraction, binarization, morphological operations, and skeletonization. The FD was then calculated using the box‐counting function of the software (Figure 4). All radiographic image acquisition, processing, scanning, ROI selection, and FD measurements were performed by the same investigator (O. G.).

**Figure 3:**
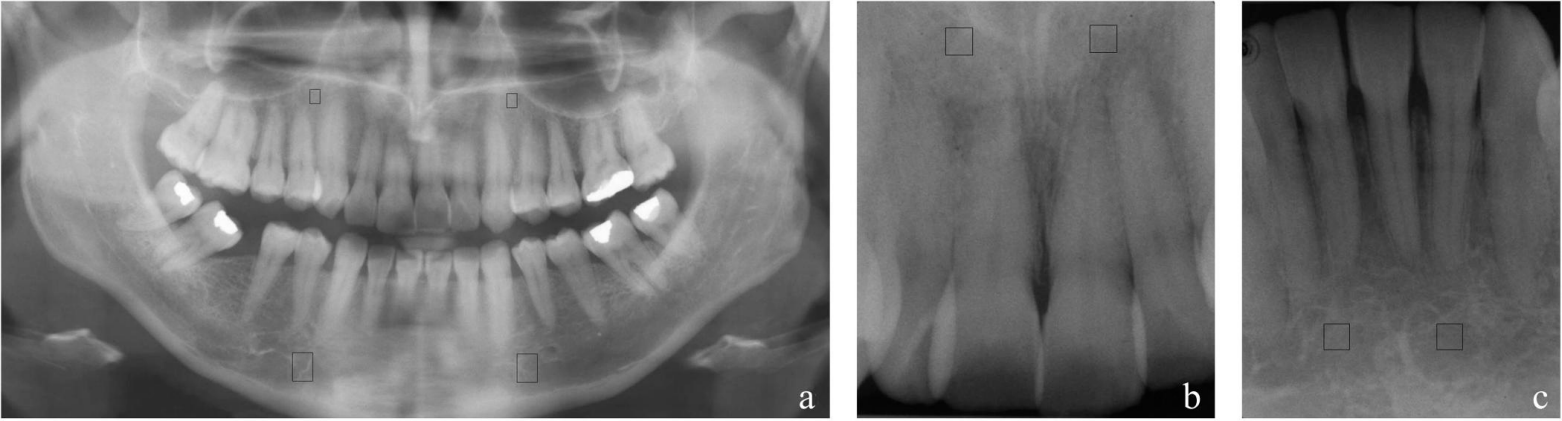
Region of interest (ROI) selection in radiographs. (a) ROIs in cropped panoramic radiograph, (b,c). ROIs in cropped periapical radiographs.

**Figure 4:**
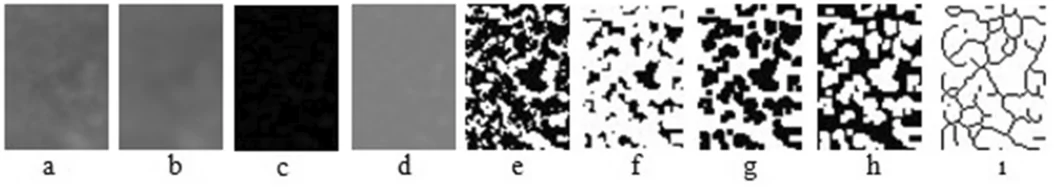
Steps of FD analysis. (a) Cropped ROI image, (b) Blurred duplicate image, (c) Subtraction of blurred image from the original, (d) Adding 128 to each pixel, (e) Thresholding at gray value 128, (f) Erosion, (g) Dilation, (h) Inversion, (i) Skeletonization.

### 2.4 Statistical Analysis

Statistical analyses were performed using IBM SPSS Statistics Version 25 (IBM Corp., Armonk, NY, USA). Generalized estimating equation (GEE) analyses were additionally performed using Python version 3.13.5 with the statsmodels package (version 0.14.6). Continuous variables are presented as mean ± standard deviation, and categorical variables as frequencies and percentages. The Pearson chi‐square test was used for categorical variables. The normality of continuous variables was assessed using the Shapiro–Wilk test. For comparisons between the fluorosis and control groups, normally distributed variables were analyzed using the independent‐samples *t*‐test, whereas variables that did not meet the normality assumption were analyzed using the Mann–Whitney *U* test. Equality of variances was assessed using Levene's test for independent‐samples *t*‐test comparisons. For comparisons between the moderate and severe fluorosis subgroups, the same statistical approach was applied according to the distribution of the data. Effect sizes were calculated using Cohen's *d* for parametric comparisons and *r* for Mann–Whitney *U* comparisons. Ninety‐five percent confidence intervals were reported for mean differences where applicable. A two‐sided *p*‐value < 0.05 was considered statistically significant. No formal correction for multiple comparisons was applied because the region‐specific analyses were exploratory and involved predefined anatomical regions. Because multiple regional FD measurements were obtained from the same participant, GEEs were additionally used to account for within‐subject correlation. Separate GEE models were constructed for panoramic and periapical radiographs using an exchangeable working correlation structure. For the fluorosis‐versus‐control comparison, fluorosis status, anatomical region, and their interaction were included in the model. For the severity analysis, fluorosis severity, anatomical region, and their interaction were included. Group‐by‐region and severity‐by‐region interactions were evaluated using joint Wald tests. Average marginal group differences across the four anatomical regions were estimated using linear contrasts of the model coefficients, with equal weighting of the four regions. Region‐specific contrasts were additionally examined to characterize differences across individual anatomical regions.

To assess intraobserver reliability, the ROI selection and complete FD measurements were repeated on 20 randomly selected participants (10 fluorosis, 10 control) 2 weeks after the initial assessment by the same examiner. Intraclass correlation coefficients (ICC) were calculated using a two‐way mixed‐effects model with absolute agreement. ICC values were interpreted as poor (< 0.50), moderate (0.50–0.75), good (0.75–0.90), or excellent (> 0.90).

## 3 Results

The study consisted of 24 individuals (12 females, 12 males) with dental fluorosis who were born and raised in Isparta, and 23 individuals (14 females, 9 males) without dental fluorosis who were not born or raised in Isparta. The mean age of the fluorosis group was 35.04 ± 11.54 years, and that of the control group was 37.91 ± 11.35 years. Statistical analysis showed no significant difference between the groups in terms of age and sex distribution (*p* > 0.05). Table 1 presents the FD values of the ROIs in panoramic and periapical radiographs for the fluorosis and control groups. In panoramic radiographs, FD values were lower in the fluorosis group than in the control group in all evaluated regions. However, the difference was statistically significant only in the 33–34 region (*p* = 0.048), with a moderate effect size. In periapical radiographs, no statistically significant differences were observed between the groups (*p* > 0.05).

**Table 1:** FD values in panoramic and periapical radiographs in the fluorosis and control groups.

Region	Fluorosis group mean ± SD	Control group mean ± SD	*p*‐value	Effect size	95% CI
*Panoramic radiograph*					
13–14 region	1.32 ± 0.06	1.35 ± 0.06	0.087	*r *= −0.25	—
23–24 region	1.33 ± 0.04	1.35 ± 0.05	0.254	*d* = −0.34	−0.04271 to 0.01158
33–34 region	1.46 ± 0.04	1.48 ± 0.03	0.048^a^	d = −0.59	−0.03904 to −0.00019
43–44 region	1.48 ± 0.05	1.50 ± 0.03	0.205	r = −0.19	—
*Periapical radiograph*					
11–12 region	1.62 ± 0.02	1.61 ± 0.02	0.282	*r *= −0.16	—
21–22 region	1.62 ± 0.02	1.62 ± 0.02	0.905	*d* = −0.04	−0.01068 to 0.00947
31–32 region	1.62 ± 0.02	1.62 ± 0.02	0.856	*d* = 0.05	−0.01014 to 0.01216
41–42 region	1.62 ± 0.02	1.62 ± 0.01	0.143	*d* = −0.44	−0.01639 to 0.00244

Abbreviations: CI, confidence interval; *d*, Cohen's *d*; *r*, effect size for the Mann–Whitney *U* test.

a Statistically significant at *p* < 0.05. Cohen's *d* was used as the effect size for independent‐samples *t*‐tests, and *r* = *Z*/√*N* was used for Mann–Whitney *U* tests. The 95% confidence intervals (CIs) represent the CIs for the mean differences in *t*‐test comparisons. — not applicable for Mann–Whitney *U* comparisons.

Because multiple regional FD measurements were obtained from the same participant, GEE analyses were additionally performed to account for within‐subject correlation. For panoramic radiographs, the average marginal FD difference between the fluorosis and control groups was statistically significant (mean difference = −0.0210, 95% CI: −0.0386 to −0.0034, *p* = 0.020), whereas the group‐by‐region interaction was not significant (*p* = 0.840), indicating no evidence that the magnitude of the group difference varied across the evaluated panoramic regions. For periapical radiographs, neither the average marginal group difference (mean difference = −0.0002, 95% CI: −0.0061 to 0.0058, *p* = 0.954) nor the group‐by‐region interaction (*p* = 0.238) was statistically significant.

In the region‐specific comparisons, FD values were significantly higher in the severe fluorosis group than in the moderate fluorosis group in the 13–14 panoramic region (*p* = 0.004) and the 11–12 periapical region (*p* = 0.019). No statistically significant differences were observed in the remaining regions (Table 2).

**Table 2:** FD values in panoramic and periapical radiographs according to fluorosis severity.

Region	Moderate fluorosis mean ± SD	Severe fluorosis mean ± SD	*p*‐value	Effect size	95% CI
*Panoramic radiograph*					
13–14 region	1.28 ± 0.06	1.36 ± 0.05	0.004^a^	*d* = −1.31	−0.11774 to −0.02499
23–24 region	1.33 ± 0.05	1.34 ± 0.03	0.435	*d* = −0.31	−0.04784 to 0.02138
33–34 region	1.47 ± 0.04	1.46 ± 0.03	0.351	*d* = 0.39	−0.01645 to 0.04437
43–44 region	1.48 ± 0.06	1.48 ± 0.04	0.862	*r* = −0.04	—
*Periapical radiograph*					
11–12 region	1.61 ± 0.02	1.63 ± 0.01	0.019^a^	*d *= −1.04	−0.02824 to −0.00287
21–22 region	1.62 ± 0.02	1.62 ± 0.02	0.570	*d* = −0.24	−0.02025 to 0.01144
31–32 region	1.61 ± 0.02	1.63 ± 0.02	0.095	*d* = −0.72	−0.02822 to 0.00242
41–42 region	1.61 ± 0.02	1.62 ± 0.02	0.441	*d* = −0.32	−0.02199 to 0.00991

Abbreviations: CI, confidence interval; d, Cohen's *d*; *r*, effect size for the Mann–Whitney *U* test.

a Statistically significant at *p* < 0.05. Cohen's d was used as the effect size for independent‐samples *t*‐tests, and *r* = *Z*/√*N* was used for Mann–Whitney *U* tests. Welch's *t*‐test was used where the equal‐variance assumption was not applied. The 95% confidence intervals (CIs) represent the CIs for mean differences in parametric comparisons. —, not applicable for Mann–Whitney *U* comparisons.

In the GEE analysis of fluorosis severity, a significant severity‐by‐region interaction was observed for panoramic radiographs (*p* = 0.004), indicating that the magnitude of the difference between the moderate and severe groups varied across panoramic regions. Accordingly, the average marginal severity effect was not statistically significant (mean difference = 0.0192, 95% CI: −0.0038 to 0.0422, *p* = 0.102). Region‐specific contrasts from the GEE model showed a significantly higher FD value in the severe fluorosis group in the 13–14 region (mean difference = 0.0714, 95% CI: 0.0301 to 0.1126, *p* < 0.001), whereas no significant differences were observed in the 23–24 (*p* = 0.406), 33–34 (*p* = 0.306), or 43–44 (*p* = 0.750) regions. For periapical radiographs, the severity‐by‐region interaction was not statistically significant (*p* = 0.464). The average marginal FD value was significantly higher in the severe fluorosis group (mean difference = 0.0097, 95% CI: 0.0010 to 0.0184, *p* = 0.028). Region‐specific GEE contrasts showed a significant difference in the 11–12 region (mean difference = 0.0156, 95% CI: 0.0045 to 0.0266, *p* = 0.006), whereas the differences in the 21–22 (*p* = 0.540), 31–32 (*p* = 0.063), and 41–42 (*p* = 0.420) regions were not statistically significant.

Intra‐observer reliability analysis demonstrated excellent agreement for the FD measurements obtained from panoramic radiographs (ICC = 0.96). For periapical radiographs, the ICC value was 0.59, indicating moderate intra‐observer reliability. Therefore, the findings obtained from periapical radiographs, particularly the subgroup comparison in the 11–12 region, were interpreted cautiously in view of the moderate measurement reliability. The relatively small sample sizes of the moderate and severe fluorosis subgroups (*n* = 13 and *n* = 11, respectively) may have limited the statistical power of subgroup comparisons. Accordingly, non‐significant findings were not interpreted as evidence of equivalence between the groups.

## 4 Discussion

A review of the literature shows that the effects of fluoride on the human body have been investigated for decades. Although fluoride is beneficial at optimal levels, excessive exposure may lead to structural alterations in bone and dental tissues (Chachra et al. 2010; Veneri et al. 2023; Han et al. 2021; Gezahegn and de Kiev 2023; Kabir et al. 2019; Wang et al. 1994; Teotia et al. 1971). Evidence specifically addressing jawbone changes in individuals with dental fluorosis remains limited. Previous fluorosis studies have primarily evaluated skeletal manifestations using radiographic examinations or bone mineral density measurements rather than FD‐based assessment (Buyukkaplan and Guldag 2012; Tamer et al. 2007; Gezahegn and de Kiev 2023; Yildiz et al. 2003; Wang et al. 1994; Teotia et al. 1971; Grandjean and Thomsen 1983; Bhatia et al. 2024). The present study evaluated FD values in selected regions of the jaws in individuals with dental fluorosis and controls, and additionally examined differences according to fluorosis severity. In the comparison between the fluorosis and control groups, a statistically significant difference was identified in one panoramic region (33–34), where FD values were lower in the fluorosis group. In the severity analysis, significantly higher FD values were observed in the severe fluorosis group in the 13–14 panoramic region and the 11–12 periapical region. These findings suggest that differences in FD may be detectable in selected jaw regions in association with dental fluorosis; however, they do not provide evidence of a generalized alteration in jawbone trabecular microarchitecture.

Radiographic manifestations of skeletal fluorosis are highly variable and may include osteosclerosis, osteoporosis, deformities, and arthritic changes (Dhar and Bhatnagar 2009; Choubisa 2022; Wang et al. 1994). Objective quantitative imaging approaches may therefore provide complementary information regarding skeletal changes associated with fluoride exposure. Fluoride incorporation into hydroxyapatite crystals leads to the formation of fluorapatite, which is less soluble and more compact. Although moderate fluoride exposure has been reported to stimulate osteoblastic activity and has previously been investigated as a potential approach for the treatment of osteoporosis, excessive fluoride exposure may disrupt bone remodeling and result in alterations in bone structure. Clinically, skeletal fluorosis may manifest as musculoskeletal pain, bone deformities, and an increased risk of fractures (Kaminsky et al. 1990; Dhar and Bhatnagar 2009; Buyukkaplan and Guldag 2012; Veneri et al. 2023; Choubisa 2022; Gezahegn and de Kiev 2023; Kabir et al. 2019; Yildiz et al. 2003). In early stages, skeletal fluorosis may be asymptomatic or associated with mild increases in bone mass, whereas more advanced disease may present with osteosclerosis, osteoporosis, arthritic changes, ligament calcification, joint restriction, limb deformities, kyphosis, muscle wasting, and neurological complications (Kaminsky et al. 1990; Dhar and Bhatnagar 2009; Veneri et al. 2023; Tamer et al. 2007; Choubisa 2022; Gezahegn and de Kiev 2023; Savas et al. 2001; Wang et al. 1994; Teotia et al. 1971; Grandjean and Thomsen 1983).

Studies from endemic regions have reported osteosclerotic and other skeletal changes (Buyukkaplan and Guldag 2012; Tamer et al. 2007; Gezahegn and de Kiev 2023; Savas et al. 2001; Yildiz et al. 2003), whereas a more recent longitudinal computed tomography‐based study has also reported findings that do not demonstrate a consistent adverse effect of fluoride exposure on bone density (Bhatia et al. 2024). These heterogeneous findings indicate that the skeletal effects of fluoride may be complex and influenced by multiple factors.

Bone mineral density measurement is widely used to assess bone status, and DEXA is considered the reference standard for clinical assessment of bone mineral density. However, it is not routinely used in dental practice (Geraets et al. 2008; Francisco et al. 2023). Computed tomography can provide quantitative information on bone density through Hounsfield unit measurements, but bone density alone does not fully characterize bone quality because trabecular organization also contributes to mechanical properties. Trabecular bone can be characterized using parameters such as trabecular number, thickness, spacing, and FD. Because conventional dental radiographs provide two‐dimensional projections of an inherently three‐dimensional trabecular network, FD analysis can be used as an imaging‐based measure of the complexity of trabecular patterns (Bollen et al. 2001; Binici and Özbay Çetiner 2024; White and Rudolph 1999; Cavalcante et al. 2022). Therefore, FD analysis was selected in the present study as a quantitative and noninvasive approach that can be applied to routine dental radiographs.

Since its introduction by White and Rudolph (White and Rudolph 1999), FD analysis has been increasingly used to investigate jawbone trabecular patterns and systemic conditions affecting bone metabolism, particularly osteoporosis (Tosoni et al. 2006; Geraets and van der Stelt 2000; Bollen et al. 2001; Binici and Özbay Çetiner 2024; White and Rudolph 1999; Yaşar and Akgünlü 2006; Geraets et al. 2008; Cavalcante et al. 2022; Carvalho et al. 2022; Gumussoy et al. 2016; Canturk et al. 2021; Altunok et al. 2024). More recent methodological research has further highlighted the importance of standardized image‐processing procedures in FD analysis. Differences in binarization and image‐processing approaches may influence FD measurements in periapical radiographs, emphasizing the need for consistent analytical protocols (da Silva et al. 2023). Recent systematic reviews have likewise reported substantial methodological heterogeneity among dental FD studies with respect to ROI selection, image processing, and FD calculation, and have noted that standardized diagnostic FD thresholds have not yet been established (Franciotti et al. 2021; Colaco et al. 2025). These observations support the use of FD as a quantitative exploratory measure of trabecular pattern complexity while highlighting the need for methodological standardization before its routine clinical application.

Trabecular bone exhibits a complex network of interconnected plates and rods with fractal characteristics. Accordingly, FD provides a quantitative descriptor of the spatial complexity of trabecular patterns in radiographic images. In general, higher FD values indicate greater complexity of the projected trabecular pattern, whereas lower values indicate a less complex pattern (Pachêco‐Pereira et al. 2019; Geraets and van der Stelt 2000; Binici and Özbay Çetiner 2024; Yaşar and Akgünlü 2006; Cavalcante et al. 2022; Carvalho et al. 2022). Previous studies have demonstrated that FD analysis can detect differences in jaw trabecular patterns in systemic skeletal conditions, particularly osteoporosis; however, the direction and magnitude of these differences have not been consistent across studies (White and Rudolph 1999; Franciotti et al. 2021). Some investigations have reported lower FD values (Carvalho et al. 2022; Southard et al. 1996) in association with reduced bone density, whereas others have reported increased (Bollen et al. 2001) or unchanged values (Tosoni et al. 2006; Yaşar and Akgünlü 2006). These discrepancies indicate that FD should not be interpreted as a simple surrogate for bone mineral density and may partly reflect differences in imaging modality, anatomical region, ROI selection, and image‐processing protocols (Tosoni et al. 2006; Geraets and van der Stelt 2000; Bollen et al. 2001; Carvalho et al. 2022). Therefore, the lower FD observed in the 33–34 region in the fluorosis group should be interpreted as a difference in radiographic trabecular pattern complexity rather than direct evidence of reduced bone quality.

Panoramic and periapical radiographs are widely available imaging methods in dentistry, and FA can be applied to these images for evaluating trabecular bone structure without additional radiation exposure (Pachêco‐Pereira et al. 2019; Tosoni et al. 2006; Bollen et al. 2001; White and Rudolph 1999; Geraets et al. 2008). In the present study, both imaging methods were used to evaluate different predefined anatomical regions. Panoramic radiographs were acquired digitally, whereas the periapical radiographs were originally acquired using an analog technique and subsequently digitized. The digitization procedure was standardized across the periapical images to reduce variability related to scanning conditions. Nevertheless, the two imaging modalities are not directly equivalent. Differences in image acquisition, spatial resolution, projection geometry, anatomical superimposition, and scanning parameters may influence image characteristics and consequently FD measurements (Bollen et al. 2001; Chen and Hollender 1995). Therefore, the findings obtained from panoramic and periapical radiographs should be interpreted separately rather than considered directly interchangeable. From a clinical perspective, the present findings do not support the use of FD analysis as a diagnostic test for skeletal or jawbone involvement in fluorosis. Rather, they demonstrate the feasibility of obtaining quantitative FD measurements from routine dental radiographs and provide preliminary data for hypothesis generation. Clinical applicability would require validation against established measures of bone density or microarchitecture, standardized imaging protocols, larger samples, and prospective or longitudinal study designs.

The moderate intra‐observer reliability observed for periapical radiographs (ICC = 0.59), compared with the excellent reliability observed for panoramic radiographs (ICC = 0.96), should also be considered when interpreting the periapical findings. Intra‐observer reliability was assessed by repeating the ROI selection and the complete FD measurement procedure. Because the subsequent image‐processing steps were performed using the same standardized protocol, variability between repeated measurements primarily reflects differences associated with ROI selection and placement. Although the same anatomical criteria were applied within each imaging modality, the periapical images provided a relatively larger area in which the ROI could be positioned. Consequently, small differences in ROI placement between repeated measurements may have contributed to measurement variability. Differences in image resolution and the digitization of analog periapical radiographs may also have contributed. However, these factors were not independently evaluated and should therefore be regarded as possible contributors rather than demonstrated causes of the lower reliability. Accordingly, the statistically significant difference observed in the 11–12 periapical region in the severity analysis should be considered exploratory and interpreted with caution.

The observed regional differences in FD cannot be attributed to a specific biological mechanism on the basis of the present data. The study did not directly assess bone turnover, bone volume, mineral composition, trabecular thickness, trabecular connectivity, or the amount and duration of fluoride exposure. Therefore, the present findings provide preliminary radiographic evidence regarding FD patterns in individuals with dental fluorosis but do not establish how fluoride exposure affects jawbone microarchitecture (Chachra et al. 2010; Gezahegn and de Kiev 2023; Wang et al. 1994; Li et al. 2023). Previous studies have demonstrated that trabecular patterns vary between anatomical regions of the jaws (Yaşar and Akgünlü 2005; Kangvansurakit 2017). Anatomical location, functional loading, cortical–trabecular relationships, and differences in ROI dimensions may influence FD measurements. However, these factors were not independently evaluated in the present study and should therefore be considered possible explanations rather than established mechanisms for the regional differences observed.

Several additional limitations should be considered. First, no a priori power analysis was performed because this was a retrospective study in which the sample size was determined by the number of eligible fluorosis cases available during the study period. The limited sample size, particularly in the moderate (*n* = 13) and severe (*n *= 11) fluorosis subgroups, may have reduced statistical power and limits the generalizability of the findings. Second, multiple anatomical regions were evaluated separately, and formal adjustment for multiple comparisons was not applied. Therefore, region‐specific statistically significant findings should be regarded as exploratory and require confirmation in larger independent samples. The inclusion of effect sizes and confidence intervals provides additional information regarding the magnitude and precision of the observed differences but does not eliminate the possibility of type I error associated with multiple testing.

In addition, potential differences in fluoride exposure after tooth development and individual or environmental factors may influence skeletal responses to fluoride. Susceptibility to fluoride‐related skeletal effects may vary according to age, sex, genetic factors, physical activity, nutritional status, renal function, hormonal status, bone characteristics, and bone remodeling rate (Chachra et al. 2010; Tamer et al. 2007; Choubisa 2022; Kabir et al. 2019; Savas et al. 2001; Wang et al. 1994). In the present study, participants with known systemic conditions or medications affecting bone metabolism were excluded. However, several potential confounding factors, including smoking, menopausal status, occlusal loading, periodontal status, post‐development fluoride exposure, and environmental or lifestyle factors, were not systematically assessed and therefore could not be included in the analysis. In addition, biochemical measures of fluoride exposure were not available. Dental fluorosis was clinically diagnosed and graded using the TFI, which provides a standardized clinical assessment of fluorosis severity but does not quantify current or cumulative systemic fluoride exposure. Accordingly, TFI severity categories should not be interpreted as direct quantitative measures of fluoride dose or systemic exposure.

Importantly, within‐subject correlation arising from repeated regional measurements was accounted for using GEE models. In the fluorosis‐versus‐control comparison, the average marginal group difference was statistically significant for panoramic but not for periapical radiographs, while the group‐by‐region interaction was not statistically significant for either modality. Thus, the significant difference observed in the 33–34 region in the region‐specific analysis should be regarded as an exploratory, region‐specific observation rather than evidence of a generalized alteration in jawbone microarchitecture. In contrast, the comparison of fluorosis severity demonstrated a significant average marginal difference for periapical radiographs. For panoramic radiographs, the significant severity‐by‐region interaction further suggested that the magnitude of the difference varied across anatomical regions. However, given the small subgroup sizes, these findings should be interpreted cautiously and require confirmation in larger samples.

Despite these limitations, the findings provide preliminary evidence that FD measurements may identify region‐specific differences in trabecular patterns in individuals with dental fluorosis. Regarding the study hypothesis, the findings therefore provide partial rather than comprehensive support for the hypothesis that FD analysis can identify differences in trabecular patterns associated with dental fluorosis. The fluorosis‐versus‐control comparison yielded a significant difference in only one panoramic region, whereas severity‐related differences were observed in selected panoramic and periapical regions. Accordingly, the findings cannot be generalized to the jaw as a whole. FD analysis may provide supplementary quantitative information regarding trabecular pattern complexity, but it should not be considered a standalone diagnostic method. Future studies with larger populations, direct assessment of systemic fluoride exposure, standardized imaging protocols, and advanced imaging techniques are needed to determine whether the observed FD differences are reproducible and clinically meaningful.

## 5 Conclusion

FD analysis revealed preliminary differences in radiographic trabecular patterns associated with dental fluorosis. In the GEE analysis, a small average difference between the fluorosis and control groups was observed on panoramic radiographs, whereas no corresponding average difference was found on periapical radiographs. Severity‐related findings also differed across the analyses, including a significant severity‐by‐region interaction on panoramic radiographs. Given the small sample size, multiple regional comparisons, and moderate intra‐observer reliability of periapical measurements, these findings should be considered exploratory. Larger studies using standardized imaging protocols and direct measures of fluoride exposure and bone status are required to confirm these findings and determine their clinical relevance.

## Author Contributions

Ozlem Gormez contributed to the conceptualization and design of the study, patient recruitment, radiographic image acquisition, image analysis, data collection and evaluation, and writing of the original manuscript. Hasan Huseyin Yilmaz contributed to the conceptualization and supervision of the study. Abdulkadir Cakir contributed to the study methodology and supervision. All authors approved the final manuscript.

## Ethics Statement

This study was approved by the Ethics Committee of Süleyman Demirel University Faculty of Medicine Clinical Research (Approval no/date: 03‐11/2011). All procedures were conducted in accordance with the Declaration of Helsinki.

## Consent

Informed consent was obtained from all participants included in the study.

## Conflicts of Interest

The authors declare no conflicts of interest.

## Data Availability

The data that support the findings of this study are available from the corresponding author upon reasonable request.
